# Behavior believability in virtual worlds: agents acting when they need to

**DOI:** 10.1186/2193-1801-2-246

**Published:** 2013-05-28

**Authors:** Nikos Avradinis, Themis Panayiotopoulos, George Anastassakis

**Affiliations:** Information Systems Laboratory, Knowledge Engineering Group, Department of Informatics, University of Piraeus, 80, Karaoli & Dimitriou Street, Athens, 185 34 Greece

**Keywords:** Behavior believability, Motivated agents, Intelligent virtual agents, Needs

## Abstract

Believability has been a perennial goal for the intelligent virtual agent community. One important aspect of believability largely consists in demonstrating autonomous behavior, consistent with the agent’s personality and motivational state, as well as the world conditions. Autonomy, on behalf of the agent, implies the existence of an internal structure and mechanism that allows the agent to have its own needs and interests, based on which the agent will dynamically select and generate goals that will in turn lead to self-determined behavior. Intrinsic motivation allows the agent to function and demonstrate behavior, even when no external stimulus is present, due to the constant change of its internal emotional and physiological state. The concept of motivation has already been investigated by research works on intelligent agents, trying to achieve autonomy. The current work presents an architecture and model to represent and manage internal driving factors in intelligent virtual agents, using the concept of motivations. Based on Maslow and Alderfer’s bio-psychological needs theories, we present a motivational approach to represent human needs and produce emergent behavior through motivation synthesis. Particular attention is given to basic, physiological level needs, which are the basis of behavior and can produce tendency to action even when there is no other interaction with the environment.

## Introduction

Ever since the early days of virtual agents, believability has been an elusive goal, pursued by researchers in the field in diverse ways and through various approaches. Believability, in the context of synthetic characters, has been defined by Bates (1994) as possessing the ability to suspend the users’ disbelief, by providing an illusion of life. In other words, believability in virtual agents is all about making the human user accept they are interacting with a living character, whose existence is consistent and coherent in the context of the virtual world it is situated in. The latter part of this definition is particularly important, as it distinguishes believability from realism, two distinct, yet closely similar terms that are often confused.

Realism refers to creating high fidelity reconstructions of the physical world. On the other hand, believability has to do with a synthetic character being consistent to essence of the entity it is supposed to embody as well the coherence of this character within the world it is situated in. As Loyall and Bates (1997) have argued, realism is nor required, neither adequate to ensure believability. A high level of realism does not necessarily imply a corresponding degree of believability; instead, as Mori (1970) has suggested, the case is often that certain levels of realism may seriously undermine the acceptance of the character as real by the audience – a concept known as the uncanny valley hypothesis.

Believability is a rather complex concept, involving diverse aspects of an intelligent virtual agent. An obvious and early explored dimension of believability refers to the agent’s visual appearance and motion. Works in the film and game industry have obviously been heavily oriented towards visual quality from early on, however the virtual world community was not late in making big advances towards this direction, with the work of Tu and Terzopoulos (1994) being a remarkable early attempt towards believable animation.

Several researchers have since argued that believability extends further than the physical properties of agents and equally involve the agent’s behavior (Lester and Stone, 1997), (Prendinger and Ishizuka, 2001), (Ortony, 2003). However, the precise meaning attributed to the word “behavior” varies among different researchers. Several works tend to focus on the behavior as a way of expression of a robotic or virtual agent’s internal state by communicative means such as gaze (Poel et al., 2009), facial expression (Sloan et al., 2009), (Malatesta et al., 2009), gesture and posture (Corradini et al., 2004), or a synthesis of multiple means (Bevacqua et al., 2007) (Niewiadomski et al., 2008). In a virtual storytelling context, works such as (Ho & Dautenhahn, 2008), (Riedl and Young, 2003,2005) argue about the narrative aspect of believability, both in terms of plot coherence and character believability. In works such as (Becker et al., 2005), (Avradinis and Aylett, 2003) and (Lim & Aylett, 2007), issues related to the importance of emotion and personality (Andre 2000) in respect to believability are discussed.

It seems, so, that there are multiple aspects of behavior believability, depending on the point of view one takes on the matter. The focus of the current work is on the *generation*, rather than the expression of believable behavior. Our approach is in line with the position of De Rosis et al. (2003), who have argued that a believable agent should act in a way consistent to its goals, its state of mind and its personality.

In this line of research, we are viewing behavior as a process of making decisions consistent to the agents’ internal state and personality as well as producing a series of corresponding primitive actions to materialize these decisions, in a way that is consistent to the agent’s physical and affective state and traits. Regardless of how actions and emotions may be expressed by means of the agent’s effectors within a virtual environment, we are interested in the underlying mechanism that allows *deciding* the appropriate thing to do, according to what the agent’s physical, emotional and mental status is at the moment, as well as materializing this decision by means of action sequences that would be plausible for the particular agent to utilize, given its personality and the holding conditions in the environment at the time of execution.

It has to be noted that behavior believability does not necessarily imply complex and highly “intelligent”, realistic behaviors; as Dautenhahn (1998) argues, it is rather a matter of enabling the virtual character to produce behavior that matches what would be expected of the user, and this is something that can be accomplished by blending together various contributing elements, such as rationality, reactivity, personality and emotion. Additionally, as argued in (Ortony, 2003) believability in the behavior of an intelligent virtual agent consists in demonstrating *coherence* in the agent’s reactions and its motivational states and *consistency* among similar kinds of situations.

### Autonomy – a prerequisite for believability

If believable behavior in virtual agents is about acting consistently to the agent’s goals, then autonomy emerges as a prerequisite. Autonomy is among the primary elements an agent situated within an environment should demonstrate according to Wooldridge and Jennings (1995), and is a concept often considered as trivial within the context of intelligent agents and confused with automation. An agent may, through clever design and programming, demonstrate behavior that seems adaptive and intelligent, without requiring external intervention in runtime. However, such an agent may only be characterized as an automated agent, rather than autonomous, if its goals are given in design time by its creator. As argued by Luck and D’Inverno (1995), providing the agent with a given set of goals to pursue, is not enough to demonstrate autonomy; a goal-directed agent is not necessarily an autonomous one, as its set of goals is given and constitutes an extrinsic source of motivation rather than an innate driving force.

True autonomy, according to researchers such as Castelfranchi (1995) or Balkenius (1993), implies self-determination and the ability to select and generate one’s own goals. This requires that the agent is equipped with its own needs and wants, that can act as intrinsic motivational mechanisms that drive the agent towards action.

In virtual worlds, and particularly persistent virtual worlds, such as online virtual communities or massive multiplayer online games, it is important for computer controlled agents (non-player characters) to be able to act on their own, rather than expect input from the user or following a pre-scripted course of action. As argued by De Sevin and Thalmann (2005), an agent in a virtual world that is programmed to react depending on the user’s input, will remain idle when no such input is present, creating a zombie effect. Even if the virtual agent is programmed to follow a scripted sequence when no other input is available, its behavior will soon start to be repetitive and trivial. This undermines the environment’s believability, as it will quickly be identified by the user and is not perceived as something consistent with a living world.

Non-human characters in virtual worlds should be able to follow their own goal agenda and function as if they were living their own lives, altering their schedule when human users interact with them. This not only requires that the agent is equipped with a personal goal agenda but also that the agent has the capability to produce its own goals in execution time. Goal generation has to be complemented with a differentiation of behavior among individual agents, consistent with their particular physical, social, metal or emotional characteristics, which constitute their virtual persona.

Embracing and extending this argument, in the present work, we suggest that a truly autonomous virtual agent should be able to function without any given goals at all, and should be able to demonstrate emergent behavior, based on its own needs and wants, as they surface due to changes in the initially aimless agent’s internal state.

Key element in this attempt is the concept of motivations, which we have in the past defined as *internal emotional*, *mental or physical* (*biological*) *processes that can produce new goals or affect an agent*’*s existing goals*, *and are themselves affected by the agent*’*s own actions*, *other agents*’ *actions*, *or environmental states* (Avradinis and Aylett, 2003). Internal states prepare the ground for intrinsic motivations to emerge and drive a virtual agent into self-propelled action, create self-determination and contribute towards the believability of the virtual agent.

### Theoretical background

#### Theories of human motivation

Theories of motivation deal with how behavior is produced, directed and energized. Motivation can be either due to external factors, or due to internal motives, namely an agent’s needs, cognitions or emotions.

One of the best known theories of human motivation is Maslow’s hierarchy of needs. Since it was first presented in (Maslow, 1943), the theory was further developed by over a course of almost thirty years into a broader theory on human behavior (Maslow, 1970), however the basic principles of the theory remained the same. According to it, human needs can be distinguished into five levels of decreasing priority. At the lowest level lie one’s *Physiological* (biological) needs-these correspond to basic needs such as hunger, thirst, need for air, sleep, sex etc. When these needs are not satisfied, they may result in emotional and physical discomfort and even threaten one’s survival.

Right above physiological needs lies the need for *Safety*, which involves not only physical safety but also occupation and financial security.

*Love and belonging* needs are highly social referring to needs such as belonging to a group, having friends, family and partner.

*Esteem* needs have both a social and a self-centered aspect. They concern self-esteem, which is related to knowledge, competence and mastery, as well as social esteem, in acknowledgement, recognition and admiration from others.

**Figure 1:**
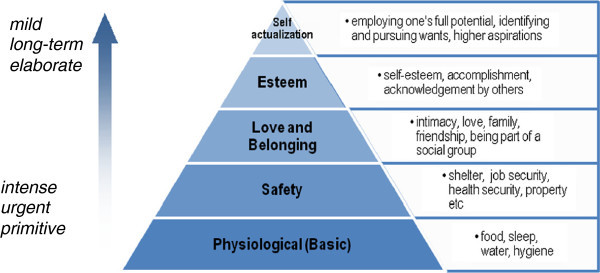
**Maslow’s hierarchy of needs.**

At the top of the pyramid lie *Self*-*Actualization* needs, a rather elusive term, which was defined as the realization of one’s full capacity and potential (Figure 1).

According to Maslow’s theory, the hierarchy is characterized by three basic principles, as follows (Reeve, 2010):

- Needs are arranged according to their urgency and their intensity; lower level needs are felt stronger and more urgently than higher level ones.
- Lower level needs appear earlier in human development.
- Needs are satisfied sequentially, one level at a time, in bottom to top order; higher level needs are not activated before lower level needs are satisfied (*a concept known as fulfillment progression*).

Needs can also be distinguished into *deficiency* ones (Reeve, and *growth*^2010^). Deficiency needs (D-needs) indicate the lack of a basic resource, or an experience; they are like internal resources that have to be replenished regularly. Failing to satisfy a deficiency need puts one in a state of deprivation, which can threaten one’s physiological or emotional state of well-being.

Growth needs (also named by Maslow as Being, or B-Needs) differ to deficiency needs, in that they do not arise because of a deprivation experience, but rather from an innate drive to evolve and develop oneself. Growth needs emerge only when all deficiency needs have been satisfied, and a need to fulfill personal potential emerges. Deficiency needs produce simpler and more stereotypical behaviors that are responses to an intense and urgent internal stimulus and aim towards satiation. Growth needs, on the other hand, are less urgent and intense but more elaborate, they can produce diverse behaviors consisting of potentially long sequences of actions and are constructive in nature. We could characterize deficiency needs as *reactive* and growth needs as *generative*.

Despite the hierarchy of needs’ age and the objections that have been risen (Wahba and Bridwell, 1976), (Alderfer, 1969), we still consider it useful, as it provides us with an

insight into the processes and the different aspects of human motivation. It also provides a good starting point for prioritizing human needs and for establishing the causality of human action, taking into account both biological as well as cognitive and affective issues. An added argument in support of the hierarchy of needs is that it is a human-centered rather than work and performance centered, as is the case with the majority of motivation theories.

**Figure 2:**
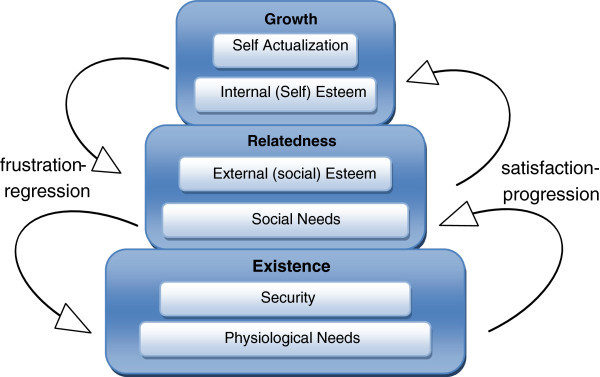
**Alderfer’s ERG theory.**

An attempt to address the shortcomings of the hierarchy of needs is Alderfer’s ERG theory (1969). Alderfer differentiated the structure of the hierarchy by splitting the Esteem needs into distinct internal (self) and external (social) esteem, resulting in 6 need categories. These 6 categories were grouped into three levels, namely Existence, Relatedness and Growth needs, hence the theories name ERG. Alderfer also contested Maslow’s strict forward progression only assumption, providing the example of an artist, who may suppress the satisfaction of lower level needs, for example social needs, or even needs at the lowest level, such as hunger or thirst, for the sake of creativity and artistic fulfillment, that at the moment consume all of the artist’s focus and energy (Figure 2).

The ERG theory clashes with Maslow’s theory in two basic issues:

- Multiple needs, belonging at different levels can be active and pursued at the same time.
- Failure to satisfy a higher level need may cause a regression to a lower level need, that is easier to satisfy (*frustration regression*).

Inspired from Maslow’s and Alderfer’s theories and drawing on previous works on motivation (Coddington and Luck, 2004), we utilize the concept of human needs as primary motivating factors to produce believable behavior. Taking up on earlier work (Avradinis and Aylett, 2003), we revisit the concept of motivations, introducing new concepts in order to integrate it into a motivated agent architecture and incorporate a layered motivation structure.

#### Needs based motivated agent approaches

The concept of the hierarchy of needs has been previously used in intelligent virtual agents, in works such as (Liu et al., 2011), (De Sevin and Thalmann, 2005), (Aydin and Orgun, 2010), (Krümpelmann et al., 2011), (Chen et al., 2003). All of them follow the generic principle of a hierarchy of needs and use similar concepts, such as basic variables, and motivations however, the techniques used for behavior productions and the architecture of each system vary.

Closest to the present work are (De Sevin and Thalmann, 2005), (Aydin and Orgun, 2010) and (Krümpelmann et al., 2011). De Sevin and Thalmann use a three-level hierarchy, distinguishing needs into Basic, Essential and Secondary. A free-flow hierarchy is followed, implying that decision making is made at an action level. Actions seem to be directly coupled to motivations, which implies that when the same motivations are active, they will produce the same behavior. Aydin and Orgun use ideas from both Alderfer’s and Maslow’s models. The authors state that they follow Alderfer’s model, although this is not consistent with the architecture, as the non-satisfaction of lower level needs is preventive for the satisfaction of higher level needs, contrary to the approach we adopt. In (Krümpelmann et al., 2011), a theoretical computational model for BDI motivated agents is presented, that follows Maslow’s theory, and implements a five-level needs hierarchy.

### The MAGE motivational synthesis model

#### Internal agent structure

The MAGE model of motivational synthesis assumes that a virtual agent’s behavior is based on the execution

of appropriate plans generated so as to fulfill the agent’s goals. These goals however, are not preassigned; instead, they are selected so as to meet the agent’s evolving needs which, in turn, depend on the agent’s internal structure.

MAGE agents maintain an *Internal Structure* as a 4-tuple : <*PA*, *AS*, *IL*, *RF*>. PA is a set of internal *Physical Attributes*. IL is a set of variables, called *Internal Levels* that represent basic parameters of the agents’ physiology. RF is a set of *Regulation Functions* governing the regulation of Internal Levels and AS is the agents’ current *Activity State*.

##### Physical attributes

We currently assume four Physical Attributes, namely, *weight*, *height*, *age* and *sex*, which, based on readings from psychology (Reeve, 2010) and physiology (Borbély and Achermann, 1999), (Mifflin et al., 1990), (Hawks et al., 2004) are among primary factors affecting the regulation of water, energy and sleep.

##### Activity state

An agent’s activity state is a measure of its energy consumption per time unit and ranges from 0, which indicates an idle state with zero energy consumption rate, to 1, which indicates a maximum energy consumption rate. Activity State does not imply a specific energy consumption rate value; it is a relative measure used for regulation purposes, as discussed below.

##### Internal levels

We also assume the existence of five Internal Levels, namely *energy level*, *water level*, *sleep reserves*, *bladder content* and *boredom*:

1. Energy level corresponds to the agent’s calorific reserves, which are decreased through agent activity and replenished by consuming food.
2. Water level corresponds to the agent’s total body water, which is again decreased through agent activity and replenished by consuming fluids or food.
3. As no solid measurement of sleep deficiency can be traced in literature, the ad-hoc concept of *sleep reserves* was introduced, as a representation of the homeostatic aspect of sleep, consistently with what is described by Borbély and Achermann (1999).
4. Bladder content corresponds to the amount of water contained in the agent’s bladder. It is reduced through excretionary behavior and is increased via a process of metabolizing body water.
5. Boredom is dependent on the agent’s motivational state; it increases while the agent remains inactive and decreases while the agent is engaged in any sort of activity.

##### Regulation functions

Internal Level values may depend on time as well as the agent’s actions and Activity State. Internal Level maxima, minima and comfort thresholds may also depend on the agent’s physical characteristics. In order to model how Internal Levels are affected by those and other factors, each Internal Level has its own set of Regulation Functions. The Regulation Functions of a particular Internal Level depend on its nature and function, in an attempt to emulate the corresponding human functions.

An extensive analysis of the regulation processes underlying each individual internal level has been given in (Avradinis et al., 2012); however, in order to provide the reader with a better understanding, the regulation of the energy level is given as an example:

As the agent consumes energy for self-preservation (even when asleep), its accumulated energy is depleted over time. The agent’s resting energy expenditure REE (or basal metabolic rate) is measured in kilocalories and can be calculated using the formula presented by Mifflin et al. (1990):

where *w* (weight) is measured in kgr, h (height) is measured in cm, and a (age) is measured in years. The consumption is then divided by the number of time quanta in a day, to calculate the agent’s instantaneous energy consumption. As this corresponds to the agent’s consumption in idle state, the current activity level must also be taken into account and is added to the idle consumption. The activity-based consumption is based on a matrix, defining five activity levels (ranging from idle to highly active) and the corresponding hourly calorie consumption for each level. Again, an instantaneous activity-based consumption is calculated and added to the idle consumption, in order to produce the total energy consumption for a particular time moment. The agent’s energy levels are replenished when the agent consumes food-the amount of calories restored to the energy levels is calculated according to the amount of food consumed and the corresponding calorie content per food/fluid unit, stored in a separate matrix.

#### Internal levels as needs

The concept of Internal Levels is particularly useful in order to model the homeostatic aspect of lower level, physiological needs, that are primarily satisfied via consummatory actions and are pure deficiency-type needs, requiring timely and urgent satisfaction. It has to be noted that internal levels are not needs themselves – it is rather the deprivation experienced because of the fall of an internal level beneath a set threshold or outside a comfort zone that causes the physical need, which in turn creates a motivation to act towards the reinstatement of equilibrium.

The basic assumptions made in the MAGE model are as follows:

- Lower level needs have an inherent priority over higher level needs.
- Needs from any level of the hierarchy, may be pursued; the existence of an unsatisfied need at a lower level is not preventive for the activation and satisfaction of another need at a higher level.
- The nature of needs may be cognitive/affective, biological or both.
- The needs placement in the hierarchy is not the only factor deciding their priority. Needs within the same level are also partially ordered, since some needs may be more urgent than others (e.g. thirst vs food, hygiene vs all the others).

#### Agent motivations

##### Motivations and needs

In the current version of the MAGE model, four basic motivations are used, corresponding to each of the basic needs: *satisfy*_*thirst*, *restore*_*energy*, *restore*_*sleep* and *void*_*bladder*.

Each motivation is represented by a variable *Motivation Intensity* value. The Intensity of a particular motivation may depend on physiological factors that may be homeostatic (based on the value of the corresponding internal level), circadian (based on the time of day), or both.

There is a natural one-to-one correspondence between needs and motivations, since a motivation essentially represents a need-driven tendency towards a particular course of action. This correspondence is manifested as an association between Internal Levels and motivations.

##### Association with internal levels

Each motivation is associated with a SetPoint Vector, which is a tuple in the form of *SP* ={*Min*, *SetPoint1*,…, *SetPointN*, *Max*}. Set points are key points in the value range of each Internal Level variable, that represent different viability zones (Meyer, 1996). Internal levels do not follow a uniform structure in respect to their set points; as each internal level may have different set points, or may have none at all. The activation of a particular need/motivation depends on whether the value of a corresponding internal level has fallen within the range of an appropriate viability zone.

Internal Level values are mapped to Motivational Intensities by the use of correlation tables, that link the internal level value to a numeric motivation intensity value and a corresponding verbal description of the feeling experienced by the agent. Correlation tables are adapted from corresponding appropriate tables reported in physiological studies.

As an example, we will again use the case of the need of hunger, represented by the motivation restore_energy. As reported in Wooley et al. (1972), the human body has no perception of the number of calories consumed to use as an estimate of its hunger; excluding other factors (such as food volume, tastes etc.,) it is rather the *belief* one has of the calorific value of consumed food that generates a sense of hunger, while the perception of satiety is usually defined based on the amount and type of food one believes can eat. A method to give a qualitative indication of the intensity of the sensation of hunger is the use of visual analogue graded scales, such as the Satiety Labeled Intensity Magnitude (SLIM) scale (Cardello et al., 2005), which is a bi-directional scale to assess satiety, based on verbal descriptions of the feeling of fullness, graded from -100 (greatest imaginable hunger) to 100 (greatest imaginable fullness) (Table 1).

**Table 1:** **The Satiety Labeled Intensity Magnitude Scale (SLIM) (adapted)**

Degree of fullness	Grade
Greatest imaginable fullness	100
Extremely full	80
Very full	75
Moderately full	45
Slightly full	30
Normal (neither hungry nor full)	0
Slightly Hungry	-20
Moderately hungry	-40
Very hungry	-55
Extremely Hungry	-65
Greatest imaginable hunger	-100

We can observe that the above approach portrays hunger as a bipolar concept, with negative values indicating states of hunger and positive values indicating fullness. At present, we only examine energy (food) balance only from a deprivation point of view and use the negative part of the scale. However, this could be further extended in the future to include the concept of fullness, which can produce behaviors avoiding (penalizing) food consumption.

For the needs of the MAGE model, we define a grading on a 0–5 basis, according to the following table, loosely based on the SLIM scale. This table is used to correlate the values of internal levels with motivation intensities (Table 2).

**Table 2:** **Intensity of satisfy**_**hunger motivation**, **corresponding energy internal level values and related feeling**

***Internal level value***	***Feeling***	***Motivation intensity***
***Energy level***	Degree of fullness	satisfy_hunger
>1*REE	Full	0
0.9*REE-1*REE	Normal (neither hungry nor full)	1
0.7*REE-0.9*REE	Slightly Hungry	2
0.5 REE-0.7*REE	Moderately hungry	3
0.3*REE-0.5*REE	Very hungry	4
0-0.3*REE	Extremely Hungry	5

##### Motivation prioritization

Each motivation is also associated with an *Aggregate Priority* value, which is calculated according to three factors: the motivation’s intensity, its *Intra*-*level priority* and its *Cross*-*level priority*.

*Intra*-*level* priority depends on the relative importance of each motivation within the same level in the hierarchy. The intra-level priority is used to represent the higher priority of water excretion, for example, relative to hunger. This is set manually in design time.

*Cross*-*level* priority is common to all motivations belonging to the same level in the hierarchy. The cross-level priority corresponds to the place of the particular need in question in the hierarchy of needs, and can be easily adapted to either correspond to ERG or Maslow levels.

The overall priority of each motivation is calculated using an aggregation function involving these three factors, which allows prioritization of one motivation over another. This is, in turn, used as a metric to evaluate which goals are selected as active.

#### Mapping needs to behaviors

In the context of the MAGE model, agent behaviors are manifested as action sequences and are triggered by goals adopted as a result of motivation variation. Goals are linked to motivations via a support/undermining mechanism, similar to the ideas presented by Coddington and Luck (2004). A goal may affect, by means of the plan that implements it, one or more motivations, potentially supporting one, while undermining another.

**Figure 3:**
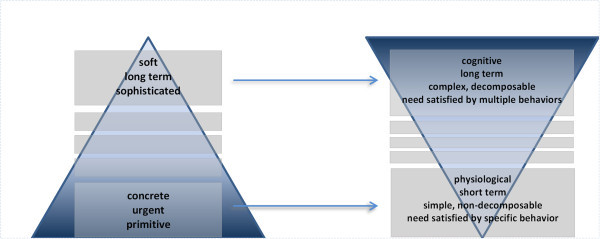
**Mapping needs to behaviors.**

The relationship between needs and the corresponding behaviors can be represented as two inverse pyramids (Figure 3). On the left, the needs' pyramid consists of multiple levels, placing primitive needs that require urgent attention at the bottom. As one progresses upwards, needs become less well-defined, more complex and with lower urgency, as usually their attention span is long term. As motivations are linked to behaviors via the support-undermine mechanism, the pyramid is inversed. Low-level needs are satisfied by mostly physiological, short term behaviors implemented either by primitive actions or by simple plans that are rarely interrupted. These behaviors are mostly mapped on a one-on-one basis to needs meaning that each need is satisfied by one behavior. Climbing up the levels of the pyramid, behaviors are growing in complexity, they require longer time to be implemented and can be decomposed into multiple subtasks. A need at higher levels may be satisfied by multiple behaviors, breaking the one-on-one need-behavior relationship of lower levels. High level behaviors may partially or fully satisfy higher level needs, over a long period of time and can be frequently interrupted, as their duration is long and their priority is low.

#### Behavior generation

Based on the above, the process of generating of a MAGE agent’s behavior can thus be outlined as follows:

1. Internal Levels are constantly adjusted according to the respective Regulation Functions, creating the effect of a constantly evolving Internal agent State.
2. Changes in the agent’s Internal State create changes to its Motivation intensities which, in turn, force the selection of goals.
3. Goal selection depends on the need each motivation aims to meet: *satisfy*_*thirst* triggers movement towards and consumption of water sources; *restore*_*energy* triggers idleness and lack of action; *restore*_*sleep* triggers a sleeping state; *void*_*bladder* triggers a suitable physiological action. Selected goals are prioritized according to the respective aggregate motivation priorities.
4. The agent commits to one of the goals selected according to goal feasibility (which is evaluated, for instance, thanks to an action planning mechanism).
5. Whenever the agent is committed to a goal, its effectors apply actions towards achieving that goal (based, for instance, on an action planning mechanism as above).

**Figure 4:**
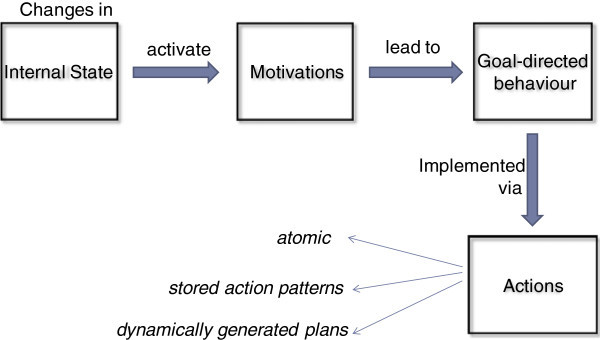
**Motivated behavior creation process in MAGE.**

The entire sequence is depicted in Figure 4.

The above process is constantly repeating in parallel with other functions the agent performs (for instance, perception and reasoning) within a complete agent architecture.

### Implementation

**Figure 5:**
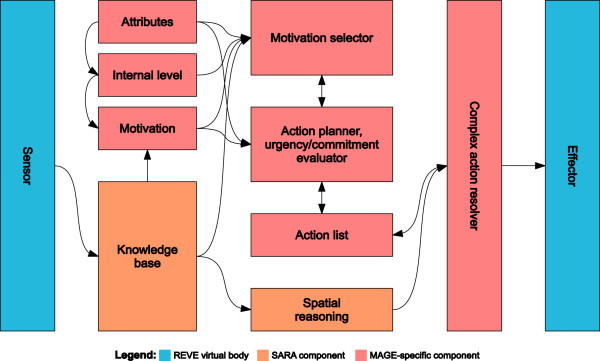
**Architectural overview of IVA based on the MAGE model.**

To evaluate the proposed model but, also, aiming towards a complete platform for experimentation and application development involving virtual agents, we have designed and implemented a Java-based prototype intelligent virtual agent (IVA) relying on the REVE platform (Anastassakis, 2010). The latter considers virtual worlds as collections of discrete virtual objects with physical properties, semantic and functionality (Anastassakis and Panayiotopoulos, 2012) and consists of a set of implemented tools and libraries for the development of intelligent virtual environment applications. The IVA’s architecture is shown in Figure 5.

The *sensor*, *effector*, *knowledge base* and *spatial reasoning* components are all provided by SARA, a Java-based library that aims to facilitate the development of REVE-compliant IVAs and is available as part of the REVE platform. Their responsibilities are, respectively, to receive perceptual data from the virtual world, to execute actions on the virtual world, to maintain, and support access to, the intelligent virtual agent’s beliefs, and to resolve complex, high-level goals with spatial references, such as path-finding, movement with respect to virtual objects, and aimless wandering.

The *attributes* component stands as a generic storage of fixed values related to the IVA’s personality and physique

(height, mass, etc.), as well as other, measurable values that may vary over time (workload, age, etc.) The *internal levels* component maintains the levels of resources accumulated with the virtual agent’s body (in particular, food and water) and enables them to change over time, according to inter-level dependencies and due to actions. It is initialized from a configuration file based on a simple specification language. The *motivation* component maintains the IVA’s collection of motivations (to satisfy hunger, thirst and boredom). Motivations are implemented as Java objects and handled as runtime plug-ins.

The *motivation selector*’s responsibility is to select a single motivation based on current attributes, internal levels, motivations and beliefs. The *action planner* takes under account a number of factors, including the selected motivation and the level of other motivations, and generates a sequence of complex actions which, when executed, are expected to serve the selected motivation. Then, it evaluates the urgency of the selected motivation; if the selected motivation is in a state of urgency – implying that the associated need or needs must be immediately satisfied – the *action list* is emptied. This essentially cancels all planned actions in favor of the selected motivation’s urgency. Then, the action planner checks if the IVA is already committed to the selected motivation (that is, checks if there already is a complex action sequence in the action list previously generated so as to serve the selected motivation) and, if not, it appends the newly-generated complex action sequence to the action list. Both the motivation selector and the action planner are also implemented as Java objects and handled as runtime plug-ins.

The *complex action resolver* component is responsible for breaking complex actions down to atomic ones which can be straightforwardly executed by the IVAs effectors

(for example: a complex action of location change involving path-finding and path-following, to a set of actions of forward motion and left/right turning of the IVAs virtual body). To that end, it frequently employs the *spatial reasoning* component which maintains an up-to-date map of the part of the virtual world known to the IVA and is capable of generating paths to target locations and performing spatial calculations of various kinds.

**Figure 6:**
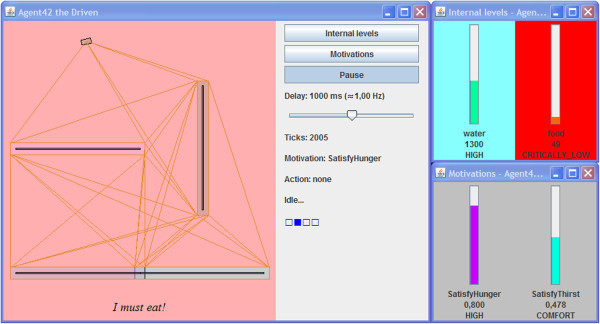
**The IVA’s user-interface.**

Figure 6 shows the IVAs user-interface containing an overview of its surroundings, a navigation mesh used for path-finding, internal levels and motivations, as well as information about the selected motivation and action list. To assist experimentation, a slider can be used to adjust the delay between operation cycles.

**Figure 7:**
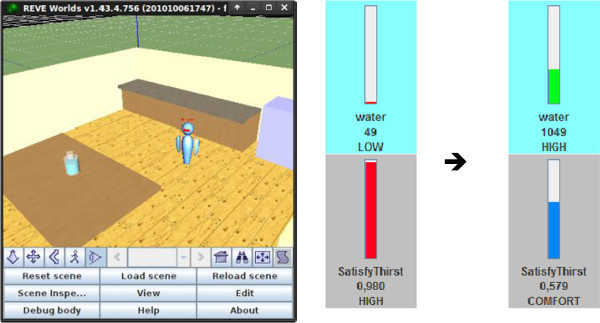
**The IVA wandering about the virtual world and consuming resources.**

The IVA’s behavior is fully motivation-driven and relies upon no hard-wired defaults. Figure 7 shows the IVA wandering around the virtual world in an effort to reduce its boredom by obtaining new knowledge about its surroundings. Various kinds of consumable resources are scattered around the virtual world, containing food (energy), water or both. Those were modeled as REVE items of a *consumable* item class equipped with custom semantic aspect annotations to represent food and water content as well as custom access aspect functions permitting them to be consumed. Figure 7 also depicts the changes in relevant internal level and motivation values that happen when the IVA consumes a bottle of 1000 ml of water in an effort to satisfy its increased thirst. The location of the water bottle had become known previously, as a result of the virtual agent’s wandering behavior, caused by an active motivation to reduce its boredom.

The above-described prototype, is fully-functional, highly-configurable and designed for extendibility. It has already proven itself capable of supporting diverse kinds of experiments through the use of various internal level specifications as well as different motivation, motivation selector and action selector plug-in implementations.

## Conclusions

A major hurdle in modeling needs as motivations was expressing vague feelings and verbal descriptions of how needs were experienced into numeric values suitable for processing. Specifically, moving from basic physiological variables (internal levels) to motivations, one soon comes across the problem of translating the concrete values of internal levels into motivation intensities. This problem was encountered with all of the basic needs. The use of graded scales from the field of psychology and physiology was of great assistance, however ad hoc assumptions and adaptations had to be made in order to produce a working model. There is definitely much room for refinement in this regard, especially as far as it concerns the concept of hunger, where the mechanism assumed in MAGE is purely short term and homeostatic and disregards long term energy storage, emotional eating or taste, hence, the approach followed for grading motivation intensity is arbitrary.

As the proposed model is still work in progress, our first empirical results are derived mostly from subjective evaluation of observed IVA behaviors. However, they are highly encouraging: We have managed to program agents capable of preserving themselves over long periods of time given unlimited resources. Accordingly, we aim to extend both the model, at a theoretical level towards increased believability, as well as the implementation, towards a complete experimentation and application development platform for IVAs encompassing the proposed model.

The concept of Physical Attributes can be extended to a broader set that include Affective Attributes as well as Mental Attributes, dependent on a Personality Model (Zoumpoulaki et al., 2010). Further work is also necessary regarding the calibration of the Internal Levels’ regulation functions, as well as the weights used to calculate the Aggregate Priorities of motivations. This task has proved to be particularly hard to tackle, as no specific link between the values of basic physiological parameters and the perceived intensity or urgency of the corresponding motivations has been found in bibliography.
